# Deformation compatibility in a single crystalline Ni superalloy

**DOI:** 10.1098/rspa.2015.0690

**Published:** 2016-01

**Authors:** Jun Jiang, Tiantian Zhang, Fionn P. E. Dunne, T. Ben Britton

**Affiliations:** 1 Department of Materials, Imperial College London, Exhibition Road, London SW7 2AZ, UK; 2 Department of Mechanical Engineering, Worcester Polytechnic Institute, 100 Institute Road, Worcester, MA 01609-2280, USA

**Keywords:** deformation gradient compatibility, geometrically necessary dislocations, single slip, high-resolution electron backscatter diffraction

## Abstract

Deformation in materials is often complex and requires rigorous understanding to predict engineering component lifetime. Experimental understanding of deformation requires utilization of advanced characterization techniques, such as high spatial resolution digital image correlation (HR-DIC) and high angular resolution electron backscatter diffraction (HR-EBSD), combined with clear interpretation of their results to understand how a material has deformed. In this study, we use HR-DIC and HR-EBSD to explore the mechanical behaviour of a single-crystal nickel alloy and to highlight opportunities to understand the complete deformations state in materials. Coupling of HR-DIC and HR-EBSD enables us to precisely focus on the extent which we can access the deformation gradient, ***F***, in its entirety and uncouple contributions from elastic deformation gradients, slip and rigid body rotations. Our results show a clear demonstration of the capabilities of these techniques, found within our experimental toolbox, to underpin fundamental mechanistic studies of deformation in polycrystalline materials and the role of microstructure.

## 1. Introduction

Characterizing and understanding the deformation behaviour of crystalline materials at microstructure scale are of great importance to further improve materials' strength and integrity performance, e.g. fracture and fatigue crack nucleation life [1–3]. These are required to design higher performance components suitable for applications in fields such as aerospace engineering and energy, structural performance and ultimately tackling societal challenges such as climate change.

Exciting progress has been made during the past 20 years in developing new characterization tools that enable us to probe microstructures with ever-more fidelity. These techniques enable us to understand the behaviour of model and industrial materials at a range of length and timescales. There are a number of examples, such as the emergence of the electron backscatter diffraction (EBSD) technique in 1990s [4], local residual stress and stored geometrically necessary dislocation density measurement using electron diffraction patterns, including high (angular)-resolution EBSD [5–7] and X-ray synchrotron techniques [8]; and local accumulated plastic slip measurement by high (spatial)-resolution digital image correlation (HR-DIC) [9–13].

These experimental techniques have arisen with a complementary array of numerical and computation tools enabling new theories to be realized in component design and engineering of real structures. This includes approaches such as finite-element crystal plasticity (CPFE) modelling [14,15], crystal plasticity fast Fourier transform (CPFFT) [16,17], molecular dynamics [18,19], and discrete dislocation dynamics [20–22]. These tools enable the discrete nature of materials to be captured at different time and length scales and, when used appropriately, enable materials scientists and engineers to test out theories often with direct comparison with experiments; and explore deformation in conditions that are inaccessible experimentally. Fundamentally, this assists in designing components based upon credible design rules and engineering know-how.

To date, this paradigm has delivered impressive gains, but as we extend our experimental and computational arsenal of techniques, there are further opportunities to refine our understanding. From an experimental perspective, new techniques and the marrying of complementary tools drives mechanistic understanding and enables improved validation/calibration of computation tools. In this paper, we highlight this opportunity by using HR-EBSD and HR-DIC on the same sample to test fundamental theories of continuum mechanics and deformation for the very first time.

According to Lee's continuum mechanics theory, ***F***=***F***^e^***F***^p^ [23], an object's deformation can be described by total deformation gradient (***F***) which consists of total strain (***E***^total^) and total rotation (***ω***^total^). The total deformation gradient (***F***) can be achieved through a plastic deformation part (***F***^p^) followed by an elastic deformation part (***F***^e^). Similarly, ***F***^e^ and ***F***^p^ are composed of elastic strain (***E***^elastic^), lattice rotation (***ω***^elastic^), plastic strain (***E***^plastic^) and continuum rotation (***ω***^plastic^). In most crystalline systems, plastic deformation is primarily caused by dislocation slip, and affected by dislocation interactions and storage. This means that dislocation-based physical mechanistic understanding can be used to update the state of these field terms. For instance, to ensure material compatibility is satisfied, curl(***F***) should be equal to zero. If follows that the presence of plastic strain gradients [24–26,27] may be explained by the presence of geometrically necessary dislocations (GNDs) that describe the closure failure of loops around either the curl of elastic deformation field or that of the plastic deformation field (for more in-depth discussion of this topic, see the pioneering work by Nye & Kroner [28,29]).

Briefly, HR-EBSD measures a change in the atomic lattice through careful analysis of electron diffraction patterns captured within the scanning electron microscope from a single sample (and therefore macroscopic strain state). Maps of diffraction patterns are captured using conventional EBSD software and analysed offline, using image correlation techniques, for pairs of diffraction patterns. A change in deviatoric strain and lattice rotation, between reference point and test point, can be measured with very high precision (approx. 1×10^−4^ in strain and approx. 1×10^−4^ rads in lattice rotation [5]). Maps of HR-EBSD results can be used to generate field plots of the elastic deformation gradients [5]. These maps form one arm of our testing strategy for Lee's theory of continuum mechanics.

Similarly, HR-DIC measures change in the surface displacements through tracking of surface features, either naturally occurring within the microstructure or added as markers. Sophisticated image processing algorithms, very similar to those used in HR-EBSD, are used to compare micrographs captured at each strain state to measure the relative displacement field between each step (for a detailed recent review, see [30]). These displacement fields, often measured only in two dimensions, can be used to calculate the total deformation gradient (***F***) between an initial, i.e. reference, state and each test state. The precision of HR-DIC is difficult to generalize as it is a trade-off tightly correlated with the region of interest/window size used for the tracking algorithm (often image correlation). Generally, a high displacement resolution requires larger regions of interest (ROIs), which in turn reduces the spatial resolution of the resultant strain field. In many circumstances, the nature of deformation at the microstructural length scale involves a compromise where the strain fields measured are largely insensitive to very small elastic strains found in metals [9,31].

Gioacchino *et al.* [11] conducted some pioneering work on developing the HR-DIC technique by depositing nano-size gold particles to achieve sub-micro spatial resolution and several other groups have also made significant contributions to the HR-DIC technique and address various plasticity problems, namely strain localization near Ni twin boundaries, strain accumulation in fatigue crack growth and damage resistance criteria in dual phase steels (e.g. [12,32–34]).

To recap, from the continuum mechanics perspective, HR-DIC measures the total deformation gradient between deformation steps, including total strain and total rotation; while HR-EBSD measures the elastic deformation gradient, including elastic strain and lattice rotation relative to a reference point within each grain for each particular deformation step.

The aim of this current work is to assess whether these two complementary techniques inform us of the complete deformation state of a crystal and aid our understanding of the validity of continuum mechanics, or more precisely kinematics, at the microstructural length scale. We focus on understanding deformation in a single crystal of an Ni superalloy at room temperature, which deforms through dislocation mediated plasticity, and enables us to explain relative contributions to the total deformation gradient and elastic deformation gradient terms which can be accessed with HR-DIC and HR-EBSD, respectively. This study complements a wider campaign of understanding deformation, plasticity and fatigue in Ni superalloys with high fidelity modelling and experimental tools (e.g. [35–37]) in order to predict fatigue crack initiation and improve lifeing strategies.

## 2. Methodology

### (a) Sample preparations and mechanical test

**Figure 1.**
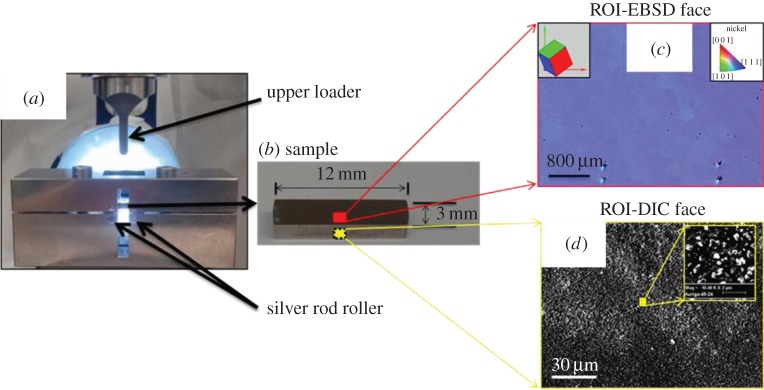
Experimental set-up. In-house made three-point bending rig (*a*). The two lower supporting silver steel rollers and an upper loader are indicated. (*b*), testing a single crystalline Ni superalloy sample with marked dimensions (12×3×3 mm) is shown as an insert in which the face and region for HR-EBSD measurement and opposite face for HR-DIC measurement are indicated as red and yellow rectangles, respectively. (*c*) An enlarged area on the EBSD face (red framed image) shows an IPF generated with respect to the horizontal direction. Unicell clearly illustrate crystallographic orientation within sample frame. The four black markers are micro-indentation markers to mark ROI (middle bottom region of the bar specimen). (*d*) An enlarged area on the DICface opposing to the EBSD face (yellow framed image) shows 3072 pixel × 2304 pixel SEM image of relatively uniform nanoparticle-coated surface. The small region was enlarged to highlight the morphology of nanoparticles (white dots). (Online version in colour.)

A single-crystal Ni superalloy sample MAR002 was deformed using a three-point bending test. The testing rig and studied areas are shown in figure 1*a*. The crystal was oriented such that a single slip system was active in the region studied (the maximum tensile strain section. This region was studied on one face with HR-EBSD and on the second (i.e. reverse face) with HR-DIC. These loading tests were performed using an interrupted loading protocol, where at each interruption the sample was removed from the bending rig and loaded into the scanning electron microscope (SEM) for imaging.

The sample was cut from a single crystal into a 12×3×3 mm^3^ testing bar and polished to a high-quality metallographic finish using gradually decreasing diamond particle size in suspension, from 10 to 3 to 1 μm, and finished by 40 min colloidal silica chemo-mechanical polishing. As revealed in figure 1*c*, an EBSD map with 20 μm step size was captured to check polishing quality and determine crystallographic orientation of the red framed image. No stretch or deformation were found in the EBSD map, and it was also confirmed that this sample was indeed single crystalline Ni with a crystallographic orientation close to [1 1 1] orientation pointing out of the free surface of sample. Furthermore, it is not unreasonable to assume that this specimen is subject to a plane stress condition near the free surface.

The opposite face was prepared for HR-DIC measurements. High fidelity DIC measurement requires good contrast and very fine patterning [38]. To achieve this, we developed a promising approach by coating the sample surface with 250 nm polishing suspension containing silica particles. The suspension was firstly diluted with distilled water at a ratio of 1 : 8. The solution was then put into an ultrasonic bath for 20 min to ensure silica particles were evenly distributed. Prepared silica solution was dropped on the ROI of the polished DIC face of the Ni sample, which was then put on a hot plate at a temperature of 150°C for 5 min to remove any additional liquid moisture. This reinforced the bonding between silica particles and the free surface of the sample and provided good stability in a vacuumed SEM chamber even under moderately deformed state.

**Figure 2.**
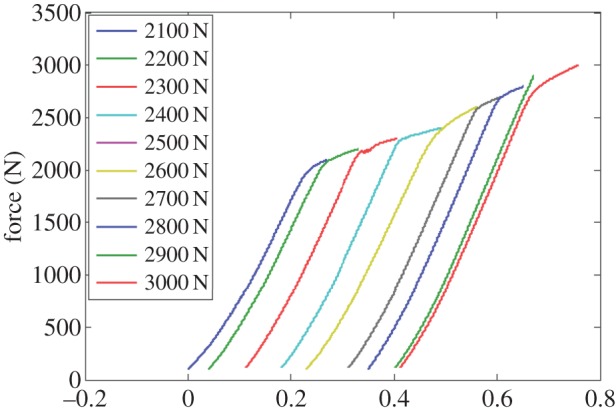
Force and displacement curves for each interrupted test, in which force was measured by 10 KN load cell and the displacement was measured from the cross-head of testing machine. The applied loading rate is 10 N s^−1^. (Online version in colour.)

This sample was inserted into the testing jig and clamped between four 3 mm diameter high strength steel rollers with a horizontal span length of 10 mm each two rollers (this geometry is optimized for push–push fatigue testing). Load was applied by an upper loader, with a diameter of 3 mm, which is connected to a conventional mechanical testing frame. The stroke and load were measured by the mechanical testing frame. The mechanical testing was performed in displacement control with target loads increasing by 100 N for each interruption, starting close to the macroscopically observed yield point. The mechanical load frame results are shown in figure 2.

After each loading increment, the sample was removed from the testing rig and examined within a Zeiss Auriga-40 SEM. Alignment of the imaged area was performed. Secondary electron images were captured at a relatively high magnification revealing an area of approximately 205×154 μm, with a pixel size of 70 nm, for DIC analysis. Images were captured at a short working distance (5 mm from the pole piece) at 20 kV and in high current mode (approx. 18 nA). The signal-to-noise ratio was improved using the line scanning average mode (two lines averaged). This results in a typical frame capture time of approximately 1.5 min per image. The imaging mode, SEM distortions and DIC analysis parameters were checked using a rigid body translation test. A root mean square (RMS) displacement error of approximately 43 nm was found [37]. These imaging conditions and relatively uniform and high-contrast patterning ensure high sensitivity of DIC measurement as shown in figure 1*d*.

The sample was flipped over and a Bruker QUANTAX EBSD system, with eFlash HR camera, was used to scan an area of 80×110 μm EBSD map for a similar region of the bending beam. EBSD was performed using similar imaging conditions to those for the DIC imaging (20 kV, high current). Patterns were captured with a step size of 0.25 μm step size, 2×2 binning (800 pixel × 574 pixel EBSPs) and 12 bit depth and saved for offline processing. The EBSD pattern frame rate was approximately 0.25 s. Each EBSD map took approximately 10 h to scan and approximately 135 Gb disc space for saving all EBSD patterns within each map.

### (b) In-house developed high-resolution digital image correlation

The digital image correlation (DIC) technique has been under development for more than two decades [9,30] and is a mature technique. The basic concept is to track features moving across the surface of two similar images using image processing techniques, such as cross-correlation, and determine the relative shifts of these features. One significant advantage of DIC is that the source of the two images is not fixed, provided that there is sufficient contrast, and therefore there is no length limit. This transfers the problem of high-resolution measurements to a matter of decorating and imaging the surface of a sample with sufficient resolution for high precision shift tracking.

In this study, we use SEM-based images which provide a high spatial resolution for sample shift determination. In our laboratory, we independently developed a DIC patterning approach using 250 nm silica particles. The obtained SEM image size is 1225×919 pixels which have a pixel size of 67 nm. We used the size of 100×100 pixels ROI with 90% overlapping in our DIC analysis, such that the spatial resolution of DIC measurement is estimated at approximately 0.7 μm. The typical measurement sensitivity using cross-correlation-based method is approximately 0.1–0.5 pixel and hence our strain resolution is lower than approximately 0.2% [13].

We have chosen to interrogate these images using an in-house developed code to ensure that our manipulation of shifts to strains is described precisely, flexibly and with maximum transparency for our calculations. This script was written within Matlab. Cross correlation was performed using two steps. The first step corrected frame average rigid body translation and far field/whole frame rotation. The second stage employed a regular grid of ROIs with size of 100 pixels and 90% overlapping for local strain measurement.

**Figure 3.**
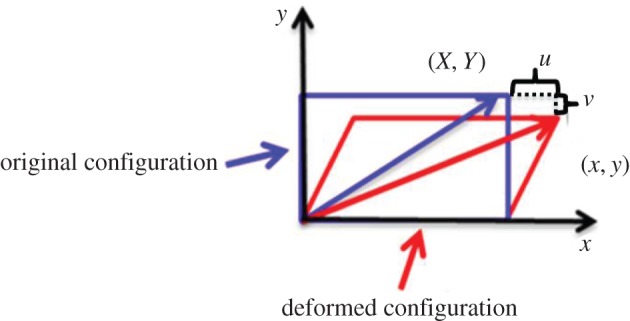
Illustrates the objects configuration changed due to deformation. Blue rectangle illustratesundeformed configuration, and red one corresponds to deformed configuration. (Online version in colour.)

The spatial intensity information within each ROI was normalized, windowed and then transformed to the Fourier domain. Band pass filtering was performed in the Fourier domain and we applied low pass (2,1) and high pass (24,12) filters to remove noise. Cross-correlation was carried out in the Fourier domain between the ROI from the first image and that from each test image. By determining the up-sampled displacement of correlation peaks, shifts along X and Y in-plane axes were measured. As illustrated in figure 3, the deformed position (*x*,*y*) of any ROI can be calculated by adding the original position (*X*,*Y*) and determined displacements *u* and *v* along the *x*- and *y*-axes, respectively.

By quadratic fitting of three neighbouring points along *x*- and *y*-axes, four of nine components of the total deformation gradient tensor ***F*** can be directly determined from DIC measurement as shown in equation (2.1). The missing five terms (shown in red) are related to the out-of-plane shifts or gradients which are inaccessible in our two-dimensional in-plane measurement.

2.1 $$\text{F}=\left(\begin{array}{ccc} F_{11} & F_{12} & F_{13}\\ F_{21} & F_{22} & F_{23}\\ F_{31} & F_{32} & F_{33} \end{array}\right)=\left(\begin{array}{ccc} \frac{dx}{dX} & \frac{dx}{dY} & \frac{dx}{dZ}\\ \frac{dy}{dX} & \frac{dy}{dY} & \frac{dy}{dZ}\\ \frac{dz}{dX} & \frac{dz}{dY} & \frac{dz}{dZ} \end{array}\right)=\left(\begin{array}{ccc} \frac{dx}{dX} & \frac{dx}{dY} & ?\\ \frac{dy}{dX} & \frac{dy}{dY} & ?\\ ? & ? & ? \end{array}\right).$$

From continuum mechanics, the total (Green) strain and rotation can be derived from ***F*** based on a polar decomposition. Here *E*_*xx*_, *E*_*xy*_, *E*_*yx*_ and *E*_*yy*_ given with respect to the global reference coordinate directions are determined directly from the following equation:

2.2 $$\text{E}=\frac{1}{2}({\text{F}}^{T}\text{F}-\text{I}),$$

where ***E*** is the Green strain tensor, ***F***^*T*^ is the transpose of ***F*** and ***I*** is the identify matrix.

The effective strain is defined as

2.3 $$E_{\mathrm{eff}}=\sqrt{\left(\frac{2}{3}\text{E}:\text{E}\right)}={\left[\frac{2}{3}(E_{11}^{2}+E_{22}^{2}+2E_{12}^{2})\right]}^{1/2}.$$

The strain sensitivity of HR-DIC is limited to approximately 0.2%, and therefore it can be reasonable and useful to consider that strains are large enough such that elastic strains are negligible compared to the plastic deformation. The three-dimensional total rotation tensor can be calculated from polar decomposition by writing the deformation gradient in terms of stretch ***U*** and rotation ***R*** as shown below

2.4 F=RU,

where ***R*** is the rotation matrix and ***U*** the symmetric matrix describing the stretch.

By rearranging equation (2.4), the total rotation ***R*** can be calculated from equation (2.5). However, the stretch matrix ***U*** is the square root of the diagonal matrix of ***F***^*T*^***F*** as shown in equation (2.6), which can be obtained by transforming coordinate system. This can be achieved by determining the eigenvalues ***U***^′2^ and eigenvectors ***Q*** of ***F***^*T*^⋅***F*** (equation (2.7)).

2.5 $$\begin{array}{rl} \text{R} & ={\text{FU}}^{-1}, \end{array}$$

2.6 $$\begin{array}{rl} {\text{F}}^{T}\text{F} & ={\text{U}}^{T}\text{U}\;\mathrm{as}\;{\text{R}}^{T}\text{R}=\text{I} \end{array}$$

2.7 $$\begin{array}{rl} \mathrm{and}\;\text{U} & ={\text{Q}}^{T}{\text{U}}^{\prime }\text{Q} \end{array}$$

where ***Q*** is the eigenvector matrix of ***F***^*T*^***F*** and ***U***′ is square root of eigenvalues of ***F***^*T*^***F***.

### (c) High-resolution electron backscattered diffraction

The high-resolution electron backscatter diffraction (HR-EBSD) technique enables calculation of the full elastic strain and lattice rotation tensors through careful analysis of two or more diffraction patterns captured within the SEM. The cross-correlation-based HR-EBSD technique was first developed by Wilkinson *et al.* in 2006 [5] and results in improved angular resolution of conventional Hough-based EBSD by a factor of 100 (approx. 4.3×10^−5^ rad compared with 4.3×10^−3^ rad [5]). Such angular resolution enabled analysis of intragranular elastic strain (i.e. stress through use of anisotropic elastic constants and Hooke's law) and higher sensitivity of geometrically necessary dislocation (GND) density measurement.

This technique continues to evolve to tackle more problems with increasing rigour. These significant improvements include robust fitting to improve its upper limit on misorientation measurement in cross-correlation [39]; as well as iterative cross-correlation to remove large rotation and rigid body translation in the first pass and reduce rotation-induced error in elastic strain measurement [40]. Further studies assessing the limits of HR-EBSD and measurements continue and include: how HR-EBSD sensitivity was affected by a range of analytical parameters were also systematically studied such as EBSD CCD detector binning size, exposure time and scanning step size [41–43]; furthermore, analysis of microstructural features such as grain boundaries necessitated an assessment of the effect of EBSD pattern overlap across grain boundaries and near microstructural features [44]. These improvements have been performed as the cross-correlation-based HR-EBSD approach has been adopted and developed by several other groups [45,46].

We only briefly describe the HR-EBSD methodology in terms of continuum mechanics and detailed descriptions of HR-EBSD methodology can be found elsewhere [6]. The basic concept of HR-EBSD is very similar to HR-DIC, in that effectively we measure the shifts of ROIs within a test image with respect to reference images within each grain. The images used here are the diffraction patterns consisting of direct projections of lattice planes and therefore we track changes within the atomic structure directly, rather than surface displacements. This means that HR-EBSD directly measures elastic deformation between points within a map. Two passes of cross-correlation approach as developed by Britton *et al.* [40] were conducted here from which the rigid pattern translation (due to pattern centre shift) and large rotations were estimated in the first pass of cross-correlation and removed. The corrected EBSD patterns were used in the second pass to re-calculate elastic strain such that elastic strains in the presence of larger lattice rotations can be calculated accurately.

**Figure 4.**
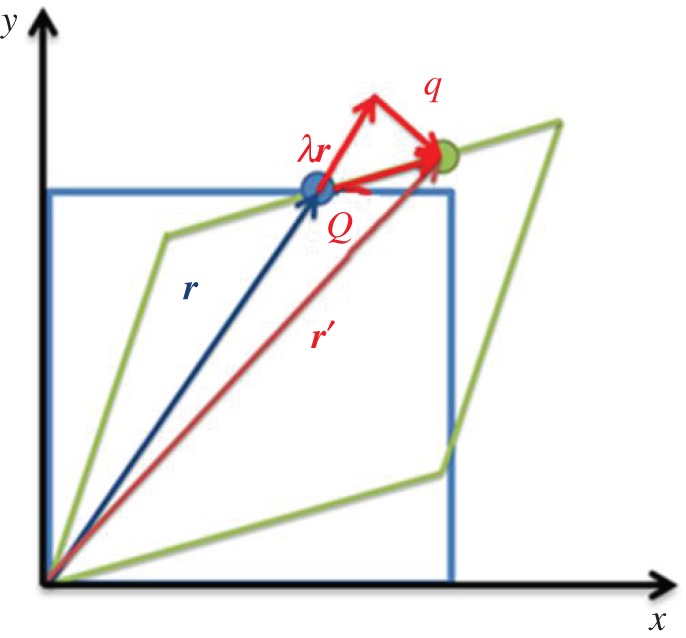
Schematically shows vector *r* in undeformed two-dimensional lattice structure (blue square) and vector *r*' in deformed (green square). Displacement dueto deformation can be expressed as *Q* and the resulted shift projected on EBSD detector is represented by *q*. (Online version in colour.)

As shown in figure 4, a reference lattice (blue square) is deformed (red square) arbitrarily. The initial vector ***r*** is distorted to ***r***′ with a displacement vector ***Q*** which is manifested as projected shift in the EBSD pattern represented by ***q***. Therefore, the vector relationship between ***q***, ***Q*** and *λ****r*** can be expressed as

2.8 q=Q−λr,

where *λ* is an unknown scalar.

Since ***Q*** can be determined by

2.9 $$\text{Q}={\text{r}}^{\prime }-\text{r}.$$

and ***r***′ can be transformed from ***r*** by elastic deformation gradient tensor ***F***^*e*^, thus

2.10 $${\text{r}}^{\prime }=({\text{F}}^{e}-\text{I})\text{r},$$

where the term ***F***^*e*^−***I*** is defined by displacement ***u***=(*u*,*v*,*w*) at position ***x***=(*X*,*Y*,*Z*) and ***I*** is the identity matrix

2.11 $${\text{F}}^{e}-\text{I}=\left(\begin{array}{ccc} \frac{du}{dX} & \frac{du}{dY} & \frac{du}{dZ}\\ \frac{dv}{dX} & \frac{dv}{dY} & \frac{dv}{dZ}\\ \frac{dw}{dX} & \frac{dw}{dY} & \frac{dw}{dZ} \end{array}\right).$$

Substituting equations (2.9) and (2.10) into equation (2.8), gives

2.12 $$\text{q}=\{{\text{F}}^{e}-\text{I}-(\lambda +1)\text{I}\}\text{r}.$$

where *λ* is an unknown scalar which cannot be obtained from shift measurement. However, it can be eliminated by substituting one of three simultaneous equations from any of the other two to yield the following two simultaneous equations:

2.13 $$r_{1}\frac{dv}{dX}+r_{2}\left[\frac{dv}{dY}-\frac{dw}{dZ}\right]+r_{3}\frac{dv}{dZ}-\frac{r_{1}r_{2}}{r_{3}}\;\frac{dw}{dX}-\frac{r_{2}^{2}}{r_{3}}\;\frac{dw}{dY}=q_{2}$$

and

2.14 $$r_{1}\left[\frac{du}{dX}-\frac{dw}{dZ}\right]+r_{2}\frac{du}{dY}+r_{3}\frac{du}{dZ}-\frac{r_{1}^{2}}{r_{3}}\;\frac{dw}{dX}-\frac{r_{2}r_{1}}{r_{3}}\;\frac{dw}{dY}=q_{1}.$$

In principle, four measurements of zone axes shifts ***q*** at widely spaced directions ***r*** within EBSD patterns are sufficient to directly calculate d*v*/d*X*,d*w*/d*X*,d*u*/d*Y*,d*w*/d*Y*,d*u*/d*Z*,d*v*/d*Z*,[d*u*/d*X*−d*w*/d*Z*],[d*v*/d*Y* −d*w*/d*Z*]. The terms d*u*/d*X*,d*v*/d*Y*,d*w*/d*Z* cannot be obtained directly because HR-EBSD does not measure hydrostatic dilation or contraction, as this does not change interplanar angles within the crystal lattice. However, as any EBSD measurement is very close to a free surface (20–50 nm) [45,47,48], it is not unreasonable to assume that the normal stress ***σ***_33_ is close to zero at the surface [49]. This assumption allows the three unknown strain terms to be determined using anisotropic Hooke's law and knowledge of the single-crystal elastic constants. We choose to apply only a ***σ***_33_ traction-free boundary condition and assess whether the out-of-plane shear stress terms are close to zero to ensure that our measurements are reasonable.

Similar to the HR-DIC approach, the measured deformation gradient tensor ***F***^e^ can be further split into elastic strain ***E***^e^ and lattice rotation ***ω***^e^ according to polar decomposition, where ***U***^e^ is the elastic stretch matrix and can be determined from the eigenvalues and eigenvector of ***F***^eT^⋅***F***^e^ according to equations (2.6) and (2.7)

2.15 $${\text{E}}^{e}=\frac{1}{2}({\text{F}}^{\mathrm{eT}}{\text{F}}^{e}-\text{I})$$

and

2.16 $$\omega^{e}={\text{F}}^{e}{\text{U}}^{e-1}.$$

It should be noted that as the deformation state of the selected reference point within a given grain is unknown, the HR-EBSD technique measures the relative elastic strain and rotation with respect to each chosen reference point. Furthermore, despite a sequential deformation series of the same crystal, it is not possible to measure with respect to an initially undeformed sample as the HR-EBSD technique measures shifts within the pattern of approximately 0.05 pixels, i.e. 2 μm in real space, which requires knowledge of the relative detector and sample precision to the same precision which is not a trivial exercise.

### (d) Crystal continuum approach for geometrically necessary dislocation density evaluation

Geometrically necessary dislocations are defined as an aggregate of dislocations which attributes to lattice geometrical shape change, e.g. a low angle grain boundary [28]. On the other hand, dislocations with opposite line directions resulting in no lattice geometrical change are described as statistically stored dislocations (SSDs) e.g. dipoles and multipoles. Strictly speaking, if circuit size is small enough to enclose a single dislocation, all such dislocations are GNDs. However, classifying dislocations as GNDs and SSDs has important advantages in continuum crystal plasticity modelling. Ashby in 1974 noticed embedded particles in matrix led to large plastic strain gradients and proposed that plastic strain gradient were due to the presence of GNDs [24].

In traditional continuum crystal plasticity modelling, length effects are not taken into account. This was changed when Fleck carried out torsion and bend experimental tests on various sizes of copper samples (12–170 μm) and found that smaller samples exhibited significantly greater hardening effect [25]. This resulted in several groups such as Fleck *et al.* [25,50] and Gao *et al.* [51,52] proposing that GNDs provided the explanation for plastic strain gradients and plastic strain-hardening effects. Hence, length-scale effects have now been introduced and successfully implemented in modern crystal plasticity finite-element modelling [15,27,53–55]. This has motivated experimental studies to measure GND density with high spatial resolution. As we cannot directly measure dislocation content across large microstructural areas [as the transmission electron microscope (TEM) requires small and thin foils to be prepared], the link between experimental measurements and GND content is necessarily indirect. We follow the routes described by Nye, Kroner and others [56,57] to briefly describe length-scale effect in crystal plasticity and outline the mathematics here for completeness gradient

In order to satisfy the compatibility condition, an integral of total deformation (***F***) around any closed loop *Γ* must equate to zero. As ***F***=***F***^e^***F***^p^, we have

2.17 $$\oint_{\Gamma }\text{F}\;dx=\oint_{\Gamma }{\text{F}}^{e}{\text{F}}^{p}\;dx=0.$$

This equation illustrates how HR-EBSD and HR-DIC measure different parts of deformation. HR-DIC measures ***F***, whereas HR-EBSD measures ***F***^e^. This demarcation will be discussed with respect to experiments shortly.

In order to access GND content, HR-EBSD measurements of ***F***^e^ can be used to obtain an integral loop to give a closure failure caused by the presence of GNDs:

2.18 $$\oint_{\Gamma }{\text{F}}^{e}\;dx=-\oint_{\Gamma }{\text{F}}^{p}\;dx\neq 0.$$

Considering an arbitrary surface, *S*, with normal ***r***, Stokes' theorem is employed to give the closure failure of a surface integral caused by the net GND density ***ρ***_G_ whose Burger's direction ***b***_G_ is parallel to ***r***.

2.19 $$\oint_{\Gamma }{\text{F}}^{e}dx=\iint_{s}\mathrm{curl}({\text{F}}^{e})\text{r}\;dS=\iint_{s}({\text{b}}_{G}⊗\rho_{G})\text{r}\;dS.$$

As dislocation density ***ρ***_G_ consists of dislocations generated from all possible slip systems *N*_*i*_ (*i*=1–12 for an Ni fcc crystal system), to simplify equation (2.19), we have

2.20 $$\sum_{i=1}^{N_{i}}{\text{b}}_{G}^{i}⊗\rho_{G}^{i}=\mathrm{curl}({\text{F}}^{e}).$$

With respect to the right-hand side of the above equation, we can express curl(***F***^e^) explicitly as

2.21 $$\mathrm{curl}({\text{F}}^{e})=\mathrm{curl}\left(\begin{array}{ccc} F_{xx}^{e} & F_{xy}^{e} & F_{xz}^{e}\\ F_{yx}^{e} & F_{yy}^{e} & F_{yz}^{e}\\ F_{zx}^{e} & F_{zy}^{e} & F_{zz}^{e} \end{array}\right)=\left(\begin{array}{ccc} \frac{\partial F_{zx}^{e}}{\partial y}-\frac{\partial F_{yx}^{e}}{\partial z} & \frac{\partial F_{xx}^{e}}{\partial z}\;-\;\frac{\partial F_{zx}^{e}}{\partial x} & \frac{\partial F_{yx}^{e}}{\partial x}-\frac{\partial F_{xx}^{e}}{\partial y}\\ \frac{\partial F_{zy}^{e}}{\partial y}-\frac{\partial F_{yy}^{e}}{\partial z} & \frac{\partial F_{xy}^{e}}{\partial z}\;-\;\frac{\partial F_{zy}^{e}}{\partial x} & \frac{\partial F_{zy}^{e}}{\partial x}-\frac{\partial F_{xy}^{e}}{\partial y}\\ \frac{\partial F_{zz}^{e}}{\partial y}-\frac{\partial F_{yz}^{e}}{\partial z} & \frac{\partial F_{xz}^{e}}{\partial z}\;-\;\frac{\partial F_{zz}^{e}}{\partial x} & \frac{\partial F_{yz}^{e}}{\partial x}-\frac{\partial F_{xz}^{e}}{\partial y} \end{array}\right).$$

Although HR-EBSD can obtain all nine components in ***F***^e^, the derivative components in equation (2.21) along surface normal *z* are inaccessible (marked as red). Thus, only three of nine components of curl(***F***^e^) can be completely and explicitly determined using the HR-EBSD technique. It is worth noting that other methods assuming negligible contribution from elastic strain enable six derivatives of the rotation field to be obtained and therefore provide six constraints [56,57].

In FCC materials, there are 12 possible slip systems, containing 12 edge dislocation types and six screw dislocation types, and only three of nine curl(***F***) terms can be completely and explicitly determined using HR-EBSD (noting that six of nine can be determined if the elastic strain contributions are ignored). This renders extracting dislocation density from individual slip systems difficult as there is an underdetermined problem to solve, which means that additional constraints must be imposed to obtain solutions [48]. Either minimization of the residual least-squares solution ‘L2’, or a L1 solution that minimizes dislocation line energy or dislocation line length is used to obtain a non-unique GND density [56]. Yet, in the experimental study to be described shortly, only a single active slip system exists; there are three equations that describe components of curl(***F***) and one unknown (the dislocation density). Thus, this becomes an overdetermined problem and we can unambiguously determine GND density for the active slip system.

For our single crystalline Ni sample, crystallographic orientation was determined by the Bruker EBSD system and described by three Euler angles (*φ*_1_,*θ*,*ψ*) as (228°, 37°, 156°). Thus its orientation matrix ***g*** was calculated as follows:

2.22 $$\begin{array}{rl} \text{g} & =\left[\begin{array}{ccc} \cos(\psi ) & \sin(\psi ) & 0\\ -\sin(\psi ) & \cos(\psi ) & 0\\ 0 & 0 & 1 \end{array}\right]\;\left[\begin{array}{ccc} 1 & 0 & 0\\ 0 & \cos(\theta ) & \sin(\theta )\\ 0 & -\sin(\theta ) & \cos(\theta ) \end{array}\right]\;\left[\begin{array}{ccc} \cos(\phi_{1}) & \sin(\phi_{1}) & 0\\ -\sin(\phi_{1}) & \cos(\phi_{1}) & 0\\ 0 & 0 & 1 \end{array}\right]\\ & =\left[\begin{array}{ccc} 0.77 & 0.55 & -0.31\\ -0.34 & 0.77 & 0.53\\ 0.53 & -0.31 & 0.79 \end{array}\right]. \end{array}$$

As DIC and EBSD maps were acquired near the central region towards the bottom tensile region of the experimental sample, over areas whose sizes are relatively small when compared with the sample size, it is not unreasonable to assume that the state of local deformation is largely uniform and uniaxial tension in nature along the horizontal direction [1 0 0] (*x*-axis). By applying Schmid's law, the primary dislocation slip system with minimum Schmid factor (*m*) was determined as follows:

2.23 m=cos(∅)cos(λ),

where *m* is the Schmid factor for all 12 possible slip systems in the FCC Ni sample, ∅ the angle between the transformed slip plane ***g***⋅{1 1 1} and applied loading direction [1 0 0] and *λ* the angle between the transformed slip direction $\text{g}\cdot \langle 1\bar{1}0\rangle$ and the loading axis.

**Figure 5.**
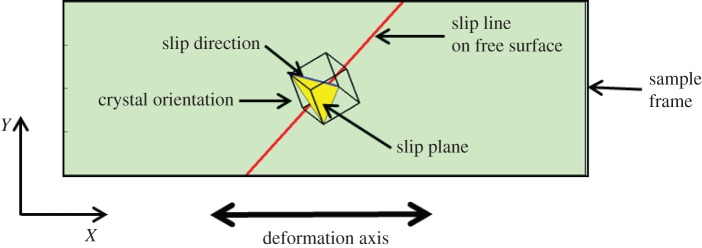
Illustration of predicted primary slip system including slip plane (yellow plane) and slip direction (blue line) in a sample frame based on Schmid factor analysis. Black unit cellshows sample's crystallographic orientation in a sample frame (DIC face), and the red line represents predicted slip line on the sample free surface. (Online version in colour.)

With the maximum value of Schmid factor *m*, the slip system with slip plane ***n*** (−1 −1 1) and slip direction ***s*** [−1 1 0] on the crystal frame was determined as the primary slip system. The slip line on the sample free surface of the DIC face was also calculated by obtaining the intersection line between the slip plane and free surface as illustrated in figure 5.

As this experiment was designed for single slip in a single crystal and the primary slip plane ***n*** and primary slip direction ***s*** were determined, these can then be substituted into the left-hand side of equation (2.20) to give

2.24 $$\sum_{i=1}^{N_{i}}{\text{b}}_{G}^{i}⊗\rho_{G}^{i}={\text{b}}_{G}^{1}⊗\rho_{G}^{1}=\frac{b_{G}}{\left|\text{s}\right|}\left(\begin{array}{c} s_{1}\\ s_{2}\\ s_{3} \end{array}\right)⊗\rho_{G}\left(\begin{array}{c} l_{1}\\ l_{2}\\ l_{3} \end{array}\right),$$

where ${\text{b}}_{G}^{1}$ is Burgers vector of the primary slip system and may be expressed as the product of half the nickel atomic spacing (*b*_G_) and slip direction vector ***s*** and divided by the magnitude of slip direction vector ***s*** in order to give a unit vector; ***ρ***_G_ is similarly the product of the geometrically necessary dislocation density magnitude (*ρ*_G_) and the dislocation line vector ***l*** which is obtained from the cross product of vectors of slip direction (***s***) and slip plane (***n***).

Substituting equations (2.21) and (2.23) into equation (2.24), we have

2.25 $$\frac{b_{G}\rho_{G}}{\left|s\right|}\left(\begin{array}{ccc} s_{1}\cdot l_{1} & s_{1}\cdot l_{2} & s_{1}\cdot l_{3}\\ s_{2}\cdot l_{1} & s_{2}\cdot l_{2} & s_{2}\cdot l_{3}\\ s_{3}\cdot l_{1} & s_{3}\cdot l_{2} & s_{3}\cdot l_{3} \end{array}\right)=\left(\begin{array}{ccc} \frac{\partial F_{zx}^{e}}{\partial y}-\frac{\partial F_{yx}^{e}}{\partial z} & \frac{\partial F_{xx}^{e}}{\partial z}\;-\;\frac{\partial F_{zx}^{e}}{\partial x} & \frac{\partial F_{yx}^{e}}{\partial x}-\frac{\partial F_{xx}^{e}}{\partial y}\\ \frac{\partial F_{zy}^{e}}{\partial y}-\frac{\partial F_{yy}^{e}}{\partial z} & \frac{\partial F_{xy}^{e}}{\partial z}\;-\;\frac{\partial F_{zy}^{e}}{\partial x} & \frac{\partial F_{zy}^{e}}{\partial x}-\frac{\partial F_{xy}^{e}}{\partial y}\\ \frac{\partial F_{zz}^{e}}{\partial y}-\frac{\partial F_{yz}^{e}}{\partial z} & \frac{\partial F_{xz}^{e}}{\partial z}\;-\;\frac{\partial F_{zz}^{e}}{\partial x} & \frac{\partial F_{yz}^{e}}{\partial x}-\frac{\partial F_{xz}^{e}}{\partial y} \end{array}\right),$$

and therefore *ρ*_G_ can, in principle, be determined unambiguously by any one of

2.26 $$\begin{array}{rl} \rho_{G} & =\frac{\left[|\text{s}|\cdot (\partial F_{yx}^{e}/\partial x-\partial F_{xx}^{e}/\partial y)\right]}{\left[b_{G}\cdot (s_{1}\cdot l_{3})\right]}, \end{array}$$

2.27 $$\begin{array}{rl} \rho_{G} & =\frac{\left[|\text{s}|\cdot (\partial F_{zy}^{e}/\partial x-\partial F_{xy}^{e}/\partial y)\right]}{\left[b_{G}\cdot (s_{2}\cdot l_{3})\right]} \end{array}$$

2.28 $$\begin{array}{rl} \text{and}\;\rho_{G} & =\frac{\left[|\text{s}|\cdot (\partial F_{yz}^{e}/\partial x-\partial F_{xz}^{e}/\partial y)\right]}{\left[b_{G}\cdot (s_{3}\cdot l_{3})\right]}. \end{array}$$

## 3. Results

### (a) Slip resulted total strain and total rotation: high spatial resolution digital image correlation

**Figure 6.**
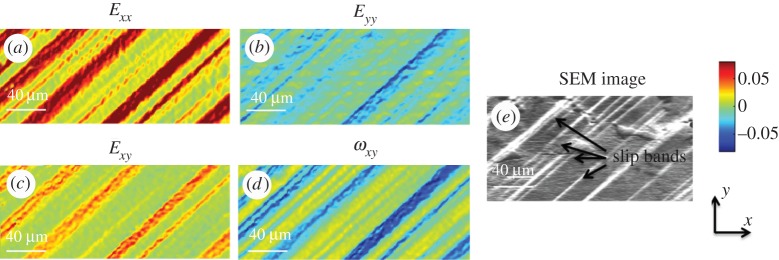
HR-DIC measured total strain and rotation components at the load of 2300 N: *E*_*xx*_ (*a*), *E*_*yy*_ (*b*) *E*_*xy*_ (*c*) and *ω*_*xy*_ (*d*) according to equations (2.2), (2.3) and (2.5). (*e*) Captured SEM image at the same magnification (1500×). The same colour bar is applied to all maps. 100 pixel × 100 pixel ROI with 90% overlap were used in DIC analysis. (Online version in colour.)

The in plane deformation gradient tensor ***F***, total strain ***E*** and total rotation ***R*** were determined using HR-DIC of SEM images after each interruption of the mechanical test, based on equations (2.1), (2.2) and (2.5), and the obtained total strain and total rotation matrix components at 2300 N are shown in figure 6. Within these data, the out-of-plane slip can be seen as topography in the SEM image, and the consequence of in-plane slip can be seen within the HR-DIC maps as shown in figure 6*e*.

The strain component *E*_*xx*_ as shown in figure 6*a* has the highest magnitude among all strain components, as this is along the direction of the macroscopic loading. By contrast, strain component *E*_*yy*_ as shown in figure 6*b* has a lower magnitude and are of opposite sign. The total rotation reveals that the crystal rotated in opposite directions between slip bands as shown in *ω*_12_ as shown in figure 6*d*.

### (b) Evolution of effective total strain: high spatial resolution digital image correlation

**Figure 7.**
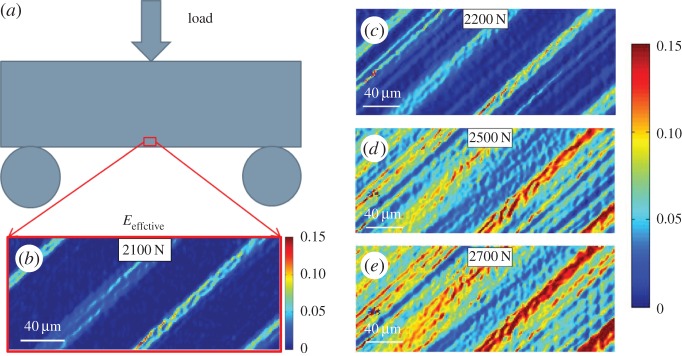
The HR-DIC measured effective strain maps as a function of applied load. (*a*) Schematically illustrates three-point bending test where red insert on bend represent HR-DIC ROI. (*b*) Total effective strain map at 2100 N load determined by equation (2.3), panels (*c*), (*d*) and (*e*) show the development of effective strain maps at 2200, 2500 and 2700 N, respectively. The sub-window size in DIC analysis was set as 100 pixel × 100 pixel with 90% overlap. (Online version in colour.)

**Figure 8.**
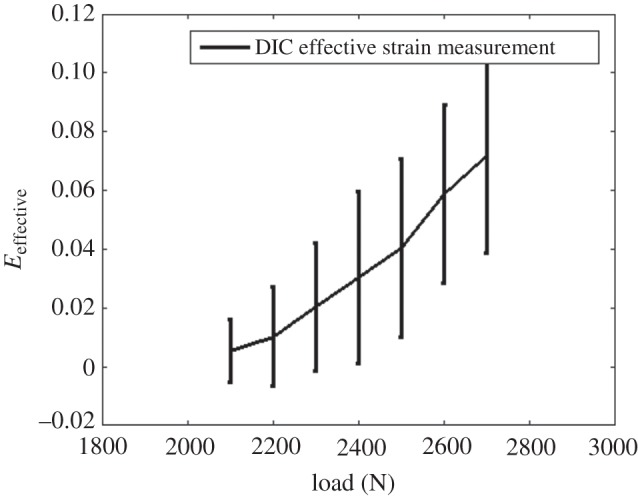
Statistical analysis of effective total strain map obtained by HR-DIC technique. The map-averaged mean values were calculated as arithmetic mean of each map and indicated as middle point of error bars. Standard deviation of maps was calculated and represented by the length of error bars.

The effective total strain was calculated to represent the strain as a scalar component and illustrates the distribution of strain caused by slip on a single slip system. Qualitative maps and quantitative assessment of effective strain have been presented in figures 7 and 8, respectively. As shown in figure 7*b*, at 2100 N loading where the sample just went beyond yield, several parallel localized bands with relatively high strain were found in the effective strain map. The direction of these slip bands agrees with the predicted slip line by Schmid factor analysis (figure 5) and matches qualitative evidence of out of plane slip shown in SEM micrographs (figure 6*e*). At this microscopic length scale, the deformation is very heterogeneous and this can also be observed within distributions of effective strain shown in figure 8. For instance, at 2500 N, the map-averaged mean value is approximately 0.04 its standard deviation has a range of approximately 0.01–0.07.

These locally resolved maps of total strain and total rotation reveal that with increasing loading, the primary slip bands were broadened. And more slip bands appeared in effective strain maps as shown in figure 7*c*–*e*. Furthermore, higher strain was accumulated along these primary slip bands. Map-averaged values and statistical distributions of the range of strain values revealed in figure 8 that the mean effective strain value and standard deviation increased with increasing loading.

### (c) Curl(*F*) and geometrically necessary dislocation density based on high spatial resolution digital image correlation measurement

To experimentally validate the geometric compatibility condition based on equation (2.17), we used DIC measured ***F*** to check whether terms within curl(***F***) would be equal to zero and thereby satisfy one of the core theories of plasticity. As only in-plane deformation gradient terms (*F*_*xx*_, *F*_*xy*_, *F*_*yy*_ and *F*_*yx*_) were measured explicitly with the HR-DIC approach, the derivative along out of plane direction Z was not available. However, we can directly determine one of nine terms of curl(***F***):curl(***F***)_*xz*_, which is sufficient to allow us to verify the compatibility condition.

**Figure 9.**
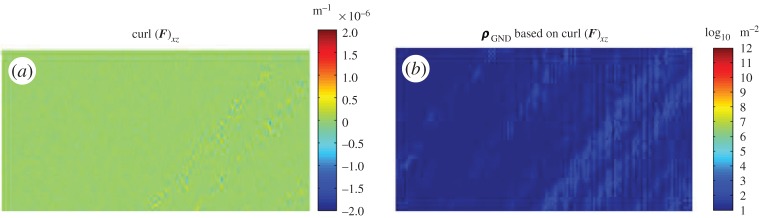
(*a*,*b*) Determined term curl(*F*)_13_ map at 2300 N and its corresponding GND density map at log 10 scale. (Online version in colour.)

The calculated curl(***F***)_*xz*_ map is shown in figure 9*a*. The majority of points have a magnitude close to 1×10^−9^ m^−1^. This is a relatively uniformly distribution and no obvious variations occurred, even in light of clear heterogeneities shown within maps of ***F*** that include significant variations due to the presence of heterogeneous deformation associated with discrete crystal slip within the slip bands. As the magnitude of curl(***F***) may be difficult to interpret, we also present the ‘effective single slip resulting GND density’ based on curl(***F***)_*xz*_ by adopting equation (2.24). This provides an easier to interpret field that can be linked with physical deformation mechanisms directly. Figure 9*b* shows estimated GND density map for which the map-averaged GND density is close to zero (5.2×10^1^ dislocation per m^2^). This value is impressively small compared with typical semiconductor silicon, which is deemed virtually defect free with a dislocation content of 1×10^8^ per m^2^ or less.

### (d) High angular resolution electron backscatter diffraction measured elastic strain and lattice rotation due to slip bands

**Figure 10.**
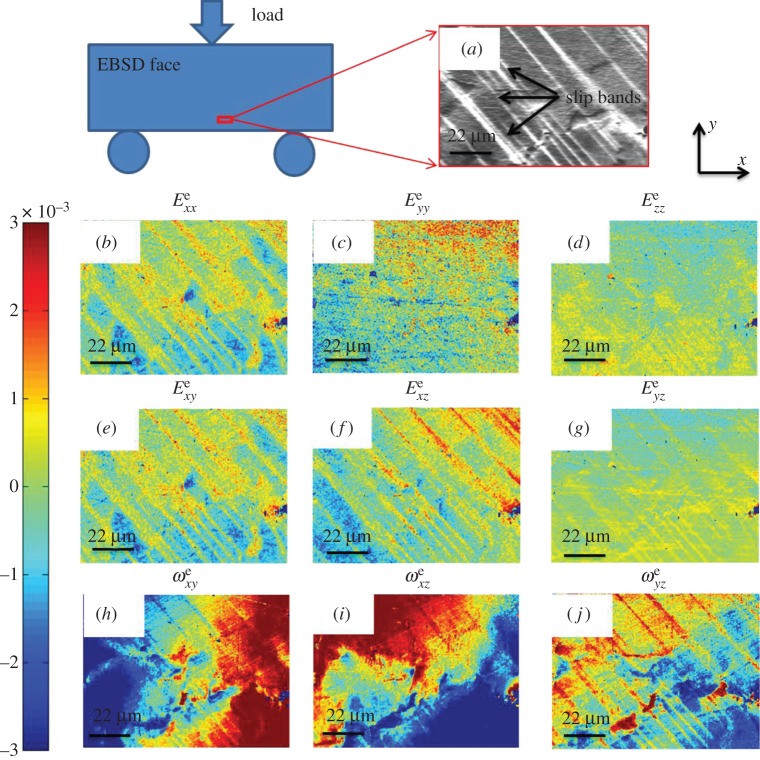
HR-EBSD measured full elastic deformation field due to slip bands at 2300 N, e.g. elastic strain maps (*b*–*g*) and lattice rotation maps (*h*–*j*) at an ROI (*a*) which is schematically shown and its corresponding SEM image is shown in (*a*). EBSD scanning step size was 0.25 μm. Owing to unknown deformation state for selected reference point, all points in elastic strain and lattice rotation map were normalized by subtracting map-averaged mean value. (Online version in colour.)****

Maps of the elastic deformation tensor terms are shown in figure 10*a*, from HR-EBSD analysis of a similar region (although on the reverse face) after the 2300 N deformation step. The reference point for each map is selected to be at the top left corner.

The HR-EBSD maps of elastic strain show the presence of the slip bands are regions of contrast on a relatively uniform background in figure 10*a*. Relatively high concentration of elastic strain $E_{xx}^{e}$, $E_{xy}^{e}$ and $E_{yz}^{e}$ are found within slip bands as shown in figure 10*b*,*e,f*. Furthermore, within the lattice rotation field there is a long-range lattice rotation gradient in $\omega_{xy}^{e}$ and $\omega_{xz}^{e}$ as shown in figure 10*h*,*i*. There is also some sharp contrast in the rotation fields associated with the presence of the slip bands in $\omega_{yz}^{e}$ as shown in figure 10*j*.

### (e) High angular resolution electron backscatter diffraction determined curl(***F***^e^) and geometrically necessary dislocation density

**Figure 11.**
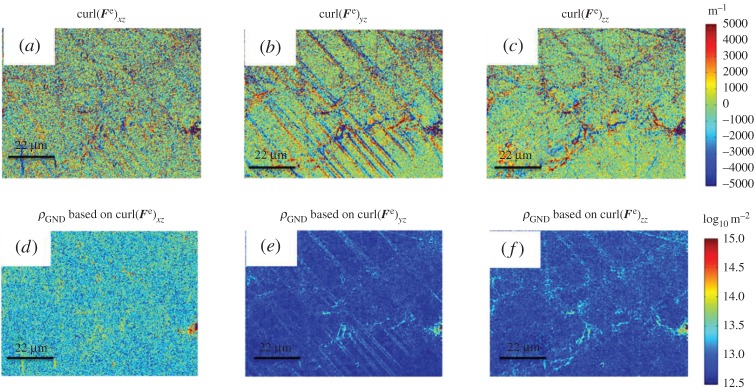
Three HR-EBSD obtained curl(*F*^e^) terms: curl(*F*^e^)_*xz*_ (*a*), curl(*F*^e^)_*yz*_ (*b*) and curl(*F*^e^)_*zz*_ (*c*) based on equation (2.21). Panels (*d*–*f*) are the corresponding GND density maps calculated based on equation (2.24)–(2.26). (Online version in colour.)

HR-EBSD was able to obtain the complete elastic deformation gradient matrix. This enables direct measurement of three complete terms of curl(***F***^e^) as shown in figure 11*a*–*c*. These three terms enable independent calculation of the GND density for single slip, as shown in figure 11*d*–*f*.

Comparison of these three curl(***F***^e^) terms (figure 11) to curl(***F***)_*xz*_ as shown in figure 9, shows that the three terms of curl(***F***^e^) in figure 11 are significantly larger and have more noise than curl(***F***)_*xz*_ (5000 to 2×10^−6^ m^−1^). All three curl(***F***^e^) terms have similar magnitude.

### (f) Evolution of elastic strain and geometrically necessary dislocation density as a function of applied load

**Figure 12.**
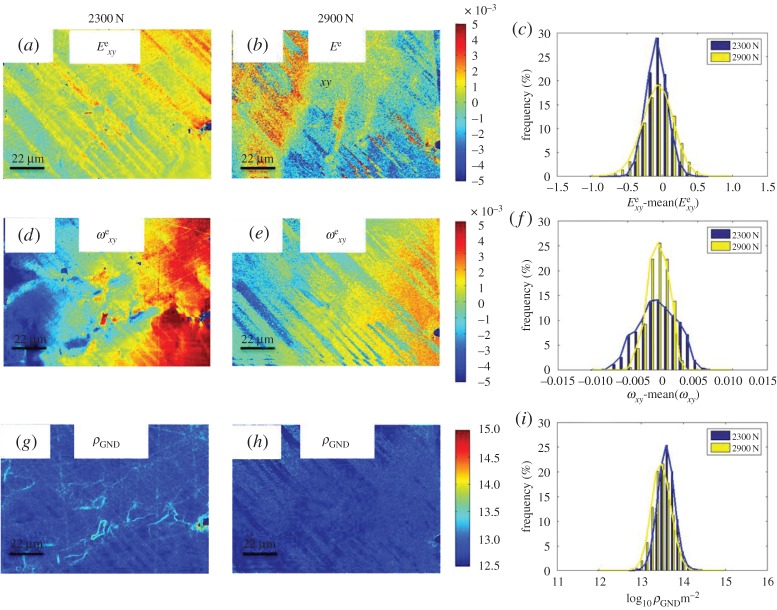
The development of elastic strain $E_{xy}^{e}$ (*a*,*b*), rotation $\omega_{xy}^{e}$ (*d*,*e*) and GND density (*g*,*h*) maps at 2300 and 2900 N. Panels (*c*,*f*,*i*) show histograms of statistical distribution and evolution of these three variables at 2300 and 2900 N, respectively. (Online version in colour.)

**Figure 13.**
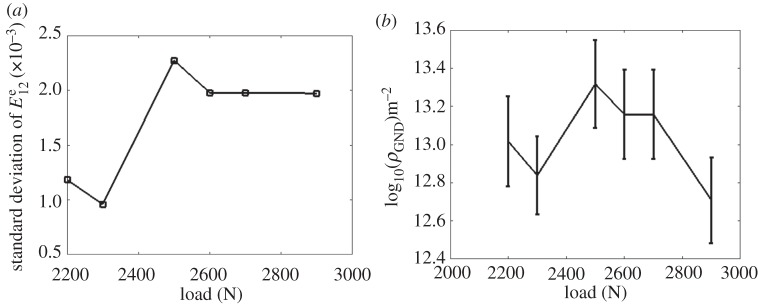
(*a*) The development of standard deviation of elastic strain component $E_{12}^{e}$ based on each map measured by the HR-EBSD technique, as a function of applied load. (*b*) The error bar plot of GND density as a function of applied load. The middle point of error bars indicates the map-averaged arithmetic mean values, and the length of error bar represents half of standard deviation for each obtained map.

For the HR-EBSD, a reference point is taken within each map. Therefore the absolute value of elastic strain is (effectively) arbitrary for comparison between loading steps. Therefore, we select to analyse the range of the distributions. As demonstrated in figures 12 and 13 there is no systematic trend in the elastic deformation fields such as elastic strain, lattice rotation and GND density at 2300 and 2900 N. This is in contrast to the total deformation measured by HR-DIC as shown in figures 7 and 8.

### (g) Comparison of total and elastic deformation: high angular resolution electron backscatter diffraction and high spatial resolution digital image correlation

**Figure 14.**
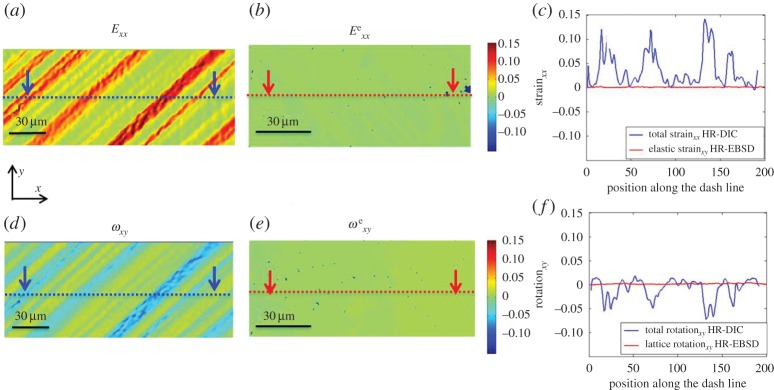
A comparison between HR-DIC obtained total deformation (total strain and total rotation) and HR-EBSD determined elastic deformation (elastic strain and lattice rotation) at 2300 N. *E*_*xx*_ (*a*,*b*) and *ω*_*xy*_ (*d*,*e*) were selected components. The same colour bar was used in all maps. Panels (*c*,*f*) show line profile comparison between total and elastic strain in *xx*-direction (*c*) and rotation in *xy*-axis (*d*). The selected lines were indicated as dash lines in (*a*,*b*,*d*,*e*). (Online version in colour.)

As the total deformation in an object is achieved by the combination of plastic deformation and elastic deformation, we compare contributions of elastic deformation to the total deformation. Figure 14 shows strain components *xx* and rotation component *xy* in both HR-DIC and HR-EBSD obtained maps at 2300 N, using the same colour scale. We can clearly see that the elastic deformation is very small compared with total deformation.

## 4. Discussion

The range of experimental techniques now available to probe deformation in crystalline materials is impressive and this provides us new insight into microstructure performance with high fidelity at a very local scale. In this study we have focused on exploring deformation during an interrupted loading campaign on an FCC Ni-based superalloy single crystal. Using HR-DIC, we have tracked the total deformation gradient, from step to step, which clearly illustrates that heterogeneous deformation occurs and that slip leads to strain localization. Analysis of the deformation fields reveals that they do indeed obey compatibility requirements as the available curl(***F***) component is zero (figure 10*a*,*b*). This improves our confidence when applying crystal plasticity simulation tools work [15,27]. Furthermore, the spatial resolution of HR-DIC implies that for some deformation mechanisms more highly refined modelling tools should be developed to accurately capture the precise crystal mechanisms at play, including slip plane spacing and saturation of plastic flow during hardening.

The complementary nature of HR-EBSD to access the elastic deformation components has been realized to assess microstructure heterogeneity at a similar length scale within the same sample (but notably on the reverse to enable ‘in parallel’ analysis). The single slip nature of this deformation simplifies our analysis significantly and affords us exquisite detail to study the precise components of the elastic strain, lattice rotation, and curl(***F***^e^) fields for further interrogation.

The elastic deformation terms, shown in figure 10, reveal significant elastic strains within the slip bands. This supports the idea that the slip bands are hardening and supporting local deformation through the presence of, perhaps, tightly bonded dipoles from expanding dislocation loops. The saturation of the slip bands, as seen within the HR-DIC maps, and subsequent activation of neighbouring slip planes implies that these dislocations contribute to local hardening within active slip planes and contribute significantly to hardening in single slip. Dislocations bonded closely together would likely result in a very small long-range strain field and minimal lattice curvature, but the spatial resolution of these HR-EBSD maps is exceptionally good (250 nm), and therefore, some highly stressed regions are observed in the elastic strain fields. These extreme values of strain, found near to dislocations stored within the crystal lattice, support analysis of extreme values of stress within polycrystal maps that are thought to be contributed from probing very close to a dislocation core and this has enabled Wilkinson *et al.* [58] to probe the total dislocation density from an interrogation of the tails of the stress distribution to a supported dislocation density.

We further note that the slip bands cause local gradients in strain and lattice rotation, which means that there are likely to be some geometrically necessary dislocations (which support these elastic strain gradients). However, we note that the curl(***F***^e^) terms that contain only elastic strain gradients are very noisy when compared with those that analyse the lattice rotation terms. This supports earlier statements [56] that in metallic systems the elastic strain gradients are usually significantly lower than the lattice rotation gradients and justifies typical assumptions regarding use of the Nye form of the dislocation density tensor, rather than the more complete Kroner form.

Longer range elastic rotations were observed in figure 10*h*,*i* were mainly due to the location of the EBSD map on the sample which was near middle and slightly skewed to the right. The map itself was not subjected to perfect tension in this region, as it is a subregion of a three-point bend sample. The strain gradient is very subtle and not visible in the long-range total strain fields (figure 8), as the elastic rotations measured from rotation of the atomic lattice are very small (figure 10).

The elastic deformation is limited by an upper bound due to the onset of plasticity. The elastic strain measurements are also taken when the system is not under external load. The elastic deformation as shown in figure 12*a*,*b* does not increase with increasing load. As our experiment was carefully designed for unconstrained single slip, there is no geometrical constraint (e.g. other slip systems, GBs) to prevent dislocations from slipping out from sample. It explains why we do not have accumulation of elastic deformation with the increasing of load.

A careful comparison of total deformation and elastic deformation is shown in figure 14, and it is found that single slip resulted in significant total strain and rotation gradient across slip bands. As total deformation consists of plastic deformation and elastic deformation, the majority part of total deformation was attributed from plasticity. This indicates that very high plastic strain gradients are present across slip bands. A casual observer could unfortunately conclude that such high plastic strain gradients would directly lead to lattice curvature and hence high GND density measured with HR-EBSD. Our experiments reveal this not to be the case (figure 12*d*–*g*).

**Figure 15.**
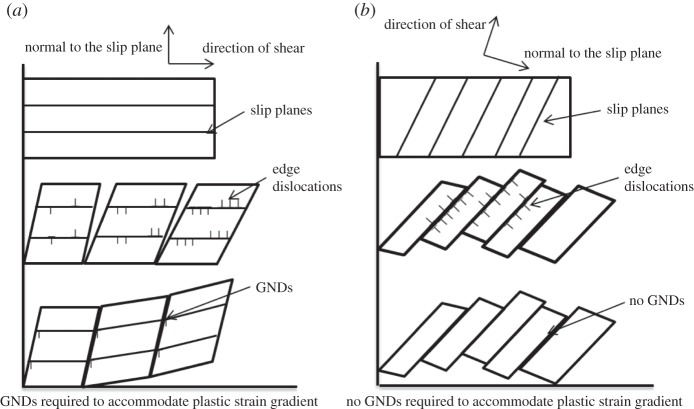
Two plastic strain gradient accommodation modes in a single slip model. (*a*) The crystal is constrained, such that GNDs are required to accommodate plastic strain gradients; (*b*) the crystal is unconstrained, such that GNDs are not necessarily needed for the resulting plastic strain gradient. Instead, plastic rotation can accommodate plastic strain gradients.

Significant total strain gradients were measured by HR-DIC across slip bands. For instance at 2200 N, slip bands has strain magnitude larger than 10% and the non-slip regions have zero strain as shown in figure 9*c*. Based on Ashby's theory [24] as shown in figure 15*a*, it would be expected that such high strain gradient (elastic strain is found to be small) would result in a significantly high GND density across slip bands. However, HR-EBSD estimated GND density cross slip bands were found to be relatively small (1×10^13^ m^−2^) as shown in figure 12*d*–*f*. This difference is interesting and requires a careful consideration of all the terms that cause changes in the total strain, ***F***.

The reason for this apparent difference is due to the nature of crystal slip and contributions to the rotation within the total deformation term. A schematic of single slip in crystalline materials is illustrated in figure 15*b*. Here we have illustrated deformation that was caused by dislocation slip on the primary slip system. Within slip bands dislocations were driven by resolved shear stress and slipped on individual slip planes. As dislocations slip out of the free surface of the sample, plastic strain was generated. However, the bonding at the atomic scale within the crystal lattice was left unchanged. This is particularly important in single crystalline materials, where there are no geometric constraints imposed unlike neighbouring grains in polycrystalline materials, and therefore the lattices were able to rotate freely. The lack of change in the local crystal lattice can clearly be observed with diffraction techniques and has been highlighted within the HR-EBSD studies here (figure 12*d*,*e*) which do not significantly show evidence of the plastic slip (in particular when compared to the topography of the slip bands or the in-plane shear as revealed with HR-DIC). The HR-EBSD most markedly reveals longer range lattice rotations across the map, which we ascribe to the bending of the three-point bend sample.

We argue that the missing link between the total deformation revealed with HR-DIC and the elastic deformation revealed with HR-EBSD is the continuum rotation of the sample due to the progressive operation of crystal slip. This results in a plastic rotation as well.

**Figure 16.**
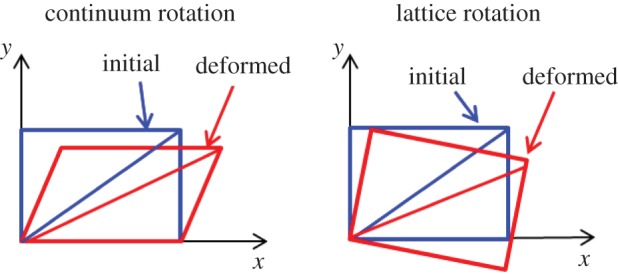
Schematic illustration of continuum rotation (plastic rotation) and lattice rotation. The blue rectangles represent undeformed crystal lattice and red rectangles corresponding todeformed lattice due to continuum rotation and lattice rotation, respectively. (Online version in colour.)

It is important to clarify that plastic rotation (continuum rotation) and the elastic rotation (lattice rotation) are very different in continuum mechanics. The total rotation can be divided into plastic and elastic rotations as shown in figure 16. If only single slip occurs as shown in figure 15*b*, lattice orientation is not necessarily changed and the frame would be rotated as shown in figure 16*a*. This type of rotation is defined usually as continuum rotation or plastic rotation. In this case, although highly localized plastic strain gradients exist, no GNDs are required to fulfil the compatibility condition. Therefore, HR-DIC observed strain gradients can be accommodated easily by continuum rotation either side of the slip band.

Where there is significant constraint, it is unlikely that the difference between HR-DIC measurements of total strain gradient and HR-EBSD measurement of elastic rotation gradient would be as marked, as the dislocations are rarely able to escape (although these dislocations will likely cause significant gradients towards the grain boundaries, rather than across individual slip bands). There could be some subtle differences that render direct comparison of HR-EBSD measured ***F***^e^ and HR-DIC ***F*** difficult. We note that, likely due to constraint, raised GND density near grain boundaries has been routinely observed in Cu with HR-EBSD [59,60].

We demonstrate that the combination of HR-DIC and HR-EBSD techniques provides unique and comprehensive information on understanding deformation processes in crystalline materials. The application of these techniques can be extended to address more complicated problems in polycrystalline materials such as ductility increase in steel through non-proportional loading (biaxial or torsion) [61,62]. The experiment must however, be carefully designed as HR-DIC is a two-dimensional surface measurement technique; and although HR-EBSD can be extended to three dimensions, extreme care must also be taken [45].

## 5. Conclusion

We have observed deformation of a single crystal of an Ni superalloy at room temperature with localized measurements of total deformation and elastic deformation with HR-DIC and HR-EBSD, respectively. This has enabled us to consider relative contributions to components of the various forms of the kinematic description of crystal deformation employed by continuum mechanics. Our most importance observation is that the so-called GNDs, which are observed in maps of elastic deformation gradient, are related to particular components of the plastic strain gradient. When crystal deformation is not constrained, significant plastic strain gradients can be observed due to the progressive operation of slip systems and dislocations emerging from the free surface. This highlights the complementary nature of experimental tools in understanding the complete deformation state of deformed materials.

In brief:

— incompatibility is not obvious in elastic deformation field: not many GNDs were required to fill slip bands (slip bands did not cause plastic strain gradients);
— long-range rotation gradients exist both in plane and out of plane captured by the position of EBSD maps;
— relatively small elastic curvature was formed adjacent to slip bands;
— localized residual stresses are present across slip bands—likely due to the storage of dipoles within the slipped bands;
— owing to the localized nature of single slip and the likely cross interaction of dislocations within the slip to form dipole structures, it is not clear if GND density increases with progressive strain during single slip; and
— for unconstrained deformation, continuum rotation dominates the total rotation and lattice rotation must be measured directly for GND analysis.

## Data accessibility

The raw data for generating figures in this paper can be found at https://zenodo.org/record/31710.
